# Aqua­(2,2′-diamino-4,4′-bi-1,3-thia­zole-κ^2^ *N* ^3^,*N* ^3′^)(thio­diacetato-κ^3^ *O*,*S*,*O*′)nickel(II) monohydrate

**DOI:** 10.1107/S1600536811015157

**Published:** 2011-05-07

**Authors:** Yan-Li Wang, Guang-Jun Chang, Bing-Xin Liu

**Affiliations:** a Department of Chemistry, Shanghai University, People’s Republic of China

## Abstract

In the title compound, [Ni(C_4_H_4_O_4_S)(C_6_H_6_N_4_S_2_)(H_2_O)]·H_2_O, the Ni^II^ cation assumes a distorted octa­hedral coordination geometry formed by a diamino­bithia­zole (DABT) ligand, a thio­diacetate (TDA) dianion and a coordinated water mol­ecule. The tridentate TDA chelates to the Ni cation in a facial configuration, and both chelating rings display the envelope conformations. The two thia­zole rings of the DABT ligand are twisted with respect to each other, making a dihedral angle of 9.96 (9)°. Extensive O—H⋯O, N—H⋯O and weak C—H⋯O hydrogen bonding is present in the crystal structure.

## Related literature

For general background to diamino­bithia­zole complexes, see: Waring (1981 ▶); Fisher *et al.* (1985 ▶). For the synthesis, see: Erlenmeyer (1948 ▶). For related structures, see: Liu *et al.* (2002 ▶).
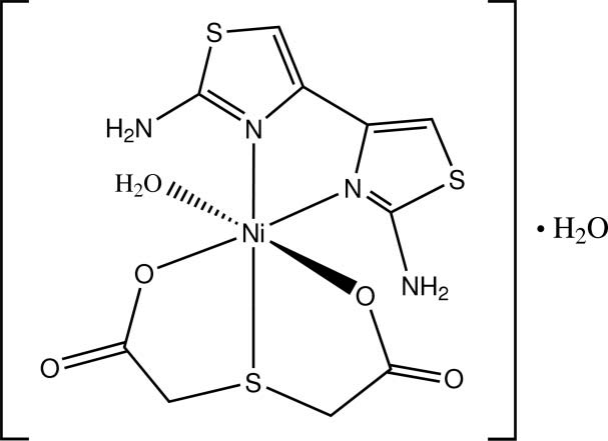


## Experimental

### 

#### Crystal data

[Ni(C_4_H_4_O_4_S)(C_6_H_6_N_4_S_2_)(H_2_O)]·H_2_O
*M* *_r_* = 441.14
Monoclinic, 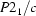

*a* = 11.856 (4) Å
*b* = 12.197 (4) Å
*c* = 12.507 (4) Å
β = 114.622 (3)°
*V* = 1644.3 (9) Å^3^
*Z* = 4
Mo *K*α radiation
μ = 1.60 mm^−1^
*T* = 295 K
0.30 × 0.24 × 0.18 mm

#### Data collection

Bruker SMART 1000 diffractometer
Absorption correction: multi-scan (*SADABS*; Bruker, 2001 ▶) *T*_min_ = 0.638, *T*_max_ = 0.750
8263 measured reflections
2888 independent reflections
2627 reflections with *I* > 2σ(*I*)
*R*_int_ = 0.016

#### Refinement

*R*[*F* ^2^ > 2σ(*F* ^2^)] = 0.022
*wR*(*F* ^2^) = 0.061
*S* = 1.06
2888 reflections
217 parameters
H-atom parameters constrained
Δρ_max_ = 0.23 e Å^−3^
Δρ_min_ = −0.38 e Å^−3^

### 

Data collection: *SMART* (Bruker, 2004 ▶); cell refinement: *SAINT* (Bruker, 2004 ▶); data reduction: *SAINT*; program(s) used to solve structure: *SIR92* (Altomare *et al.*, 1993 ▶); program(s) used to refine structure: *SHELXL97* (Sheldrick, 2008 ▶); molecular graphics: *ORTEP-3 for Windows* (Farrugia, 1997 ▶); software used to prepare material for publication: *WinGX* (Farrugia, 1999 ▶).

## Supplementary Material

Crystal structure: contains datablocks I, global. DOI: 10.1107/S1600536811015157/xu5191sup1.cif

Structure factors: contains datablocks I. DOI: 10.1107/S1600536811015157/xu5191Isup2.hkl

Additional supplementary materials:  crystallographic information; 3D view; checkCIF report

## Figures and Tables

**Table 1:** Selected bond lengths (Å)

Ni—O1	2.0763 (14)
Ni—O21	2.0944 (14)
Ni—O23	2.0357 (14)
Ni—N11	2.0634 (15)
Ni—N13	2.1094 (16)
Ni—S21	2.4461 (7)

**Table 2:** Hydrogen-bond geometry (Å, °)

*D*—H⋯*A*	*D*—H	H⋯*A*	*D*⋯*A*	*D*—H⋯*A*
O1—H1*A*⋯O1*W*	0.85	1.91	2.7540	173
O1—H1*B*⋯O22^i^	0.79	1.97	2.7399	162
N12—H12*A*⋯O24^ii^	0.81	2.09	2.8785	166
N12—H12*B*⋯O23	0.87	2.09	2.8807	152
N14—H14*A*⋯O21^i^	0.83	2.20	2.9378	149
O1*W*—H1*WA*⋯O24^i^	0.85	2.01	2.8616	177
O1*W*—H1*WB*⋯O22^iii^	0.84	1.91	2.7493	173

Symmetry codes: (i) 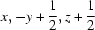; (ii) 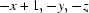; (iii) 
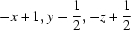
.

**Fig. 1.**
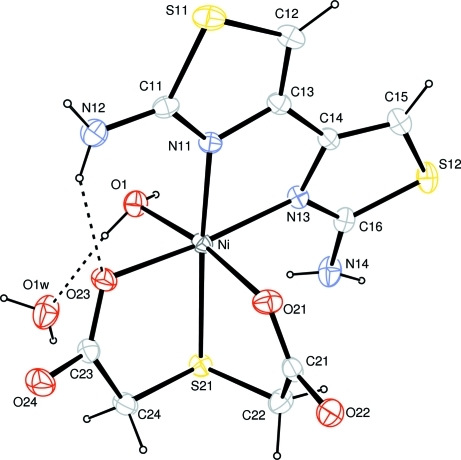
The molecular structure of (I) with 30% probability displacement ellipsoids, dashed lines showing hydrogen bonding.

**Fig. 2.**
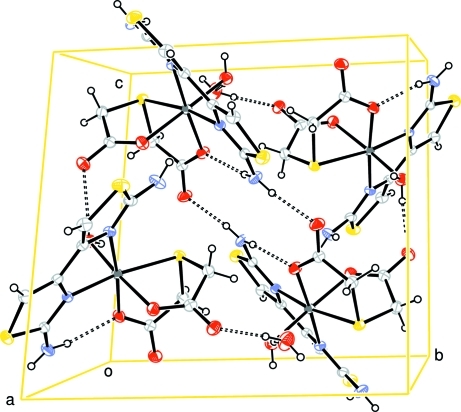
A molecular packing diagram, dashed lines showing the hydrogen bonding between NiII complex molecules.

## supplementary crystallographic information

### Comment

Transition metal complexes of DABT have shown potential application in some fields, such as the effective inhibitors of DNA synthesis of the tumor cells (Waring, 1981; Fisher *et al.*, 1985). As part of a series of investigations of metal complexes of DABT, the title Ni^II^ complex, (I), was prepared in the laboratory and its X-ray structure is reported here.

The molecular structure of the title compound is shown in Fig. 1. The complex has a distorted octahedral coordination geometry formed by one of DABT, one of TDA and one of coordinated water.

The tridentate TDA chelates to Ni^II^ atom in an envelope configuration. Two carboxyl groups of TDA monodentately coordinate to the Ni^II^ atom. Uncoordinated carboxyl oxygen atoms O22 and O24 are hydrogen bonded to the hydrogen atoms of coordinated water of the neighboring complex molecule, as shown in Fig. 2 and Table 1. Othervize, coordinated carboxyl oxygen atom O1 is hydrogen bonded to the hydrogen atoms of lattice watter molecule of the neighboring complex molecule and within complex respectively. The atom O22 and O24 hydrogen bonnded to amino group of DABT of neighboring complex and lattice water within complex respectively.

The DABT chelates to the Ni^II^ atom with a near coplanar configuration, the angle of two thiazole rings being 9.96 (11) °. The bond distance 1.462 (3) Å of C13—C14 correspond to C—C single bonds between sp2-hybridized C atoms (C—C = 1.483 Å). The *C*—*N*(amino) bond (C11—N12 = 1.323 (2) Å) distance is shorter than 1.354 (2) Å in free DABT and the other one (C16—N14 =1.353 (3) Å) distance is similar to 1.354 (2) Å in free DABT (Liu *et al.*, 2002). The C—N bond within thiazole ring one distance (C11—N11 = 1.323 (2) Å) is longer than 1.309 (2) Å and the other one (C16—N13 = 1.313 (2) Å) is similar to 1.309 (2) Å in free DABT within thiazole rings (Liu *et al.*, 2002), this suggests the existence of electron delocalization within one thiazole ring of DABT.

### Experimental

The DABT was prepared according to the literature (Erlenmeyer, 1948). An aqueous solution (20 ml) containing DABT (1 mmol) and NiCl_2_ (1 mmol) was mixed with an aqueous solution (10 ml) of thiodiacetic acid (1 mmol) and NaOH (2 mmol). The mixture was refluxed for 5 h. The solution was filtered after cooling to room temperature. Green single crystals were obtained from the filtrate after 7 d.

### Refinement

H atoms on carbon atoms were placed in calculated positions, with C—H distances = 0.93 Å (thiazole ring), and were included in the final cycles of refinement in riding mode with *U*_iso_(H) = 1.2*U*_eq_(C). Amino H atoms and water H atoms were located in a difference Fourier map and included in the structure factor calculations with fixed positional, and *U*_iso_(H) = 1.2*U*_eq_(N) and 1.5*U*_eq_(O).

### Crystal data

**Table:**

[Ni(C_4_H_4_O_4_S)(C_6_H_6_N_4_S_2_)(H_2_O)]·H_2_O	*F*(000) = 904
*M**_r_* = 441.14	*D*_x_ = 1.782 Mg m^−^^3^
Monoclinic, *P*2_1_/*c*	Mo *K*α radiation, λ = 0.71073 Å
Hall symbol: -P 2ybc	Cell parameters from 2650 reflections
*a* = 11.856 (4) Å	θ = 2.0–25.0°
*b* = 12.197 (4) Å	µ = 1.60 mm^−^^1^
*c* = 12.507 (4) Å	*T* = 295 K
β = 114.622 (3)°	Prism, green
*V* = 1644.3 (9) Å^3^	0.30 × 0.24 × 0.18 mm
*Z* = 4	

### Data collection

**Table:**

Bruker SMART 1000 diffractometer	2888 independent reflections
Radiation source: fine-focus sealed tube	2627 reflections with *I* > 2σ(*I*)
graphite	*R*_int_ = 0.016
ω scans	θ_max_ = 25.0°, θ_min_ = 2.5°
Absorption correction: multi-scan (*SADABS*; Bruker, 2001)	*h* = −14→14
*T*_min_ = 0.638, *T*_max_ = 0.750	*k* = −10→14
8263 measured reflections	*l* = −14→14

### Refinement

**Table:**

Refinement on *F*^2^	Primary atom site location: structure-invariant direct methods
Least-squares matrix: full	Secondary atom site location: difference Fourier map
*R*[*F*^2^ > 2σ(*F*^2^)] = 0.022	Hydrogen site location: inferred from neighbouring sites
*wR*(*F*^2^) = 0.061	H-atom parameters constrained
*S* = 1.06	*w* = 1/[σ^2^(*F*_o_^2^) + (0.0349*P*)^2^ + 0.4782*P*] where *P* = (*F*_o_^2^ + 2*F*_c_^2^)/3
2888 reflections	(Δ/σ)_max_ = 0.001
217 parameters	Δρ_max_ = 0.23 e Å^−^^3^
0 restraints	Δρ_min_ = −0.38 e Å^−^^3^

### Special details

**Table:**

Geometry. All e.s.d.'s (except the e.s.d. in the dihedral angle between two l.s. planes) are estimated using the full covariance matrix. The cell e.s.d.'s are taken into account individually in the estimation of e.s.d.'s in distances, angles and torsion angles; correlations between e.s.d.'s in cell parameters are only used when they are defined by crystal symmetry. An approximate (isotropic) treatment of cell e.s.d.'s is used for estimating e.s.d.'s involving l.s. planes.
Refinement. Refinement of *F*^2^ against ALL reflections. The weighted *R*-factor *wR* and goodness of fit *S* are based on *F*^2^, conventional *R*-factors *R* are based on *F*, with *F* set to zero for negative *F*^2^. The threshold expression of *F*^2^ > σ(*F*^2^) is used only for calculating *R*-factors(gt) *etc*. and is not relevant to the choice of reflections for refinement. *R*-factors based on *F*^2^ are statistically about twice as large as those based on *F*, and *R*- factors based on ALL data will be even larger.

### Fractional atomic coordinates and isotropic or equivalent isotropic displacement parameters (Å^2^)

**Table:**

	*x*	*y*	*z*	*U*_iso_*/*U*_eq_	
Ni	0.70472 (2)	0.163736 (19)	0.32141 (2)	0.02339 (9)	
O21	0.74187 (13)	0.28151 (11)	0.21850 (12)	0.0346 (3)	
O22	0.73617 (12)	0.45346 (12)	0.16017 (12)	0.0365 (3)	
O23	0.53565 (12)	0.13917 (11)	0.18614 (12)	0.0328 (3)	
O24	0.37049 (13)	0.21909 (12)	0.04991 (12)	0.0422 (4)	
O1	0.64613 (13)	0.06045 (11)	0.42019 (12)	0.0353 (3)	
H1A	0.5731	0.0782	0.4093	0.053*	
H1B	0.6862	0.0612	0.4894	0.053*	
N11	0.79168 (13)	0.04751 (12)	0.26220 (13)	0.0244 (3)	
N12	0.63575 (15)	−0.03738 (15)	0.09932 (14)	0.0364 (4)	
H12A	0.6217	−0.0852	0.0508	0.044*	
H12B	0.5822	0.0034	0.1111	0.044*	
N13	0.88791 (14)	0.17581 (12)	0.45192 (13)	0.0258 (3)	
N14	0.88728 (17)	0.30143 (15)	0.59448 (16)	0.0419 (4)	
H14A	0.8266	0.2753	0.6018	0.050*	
H14B	0.9332	0.3406	0.6512	0.050*	
S11	0.87354 (5)	−0.09125 (4)	0.15646 (5)	0.03800 (14)	
S12	1.10452 (5)	0.24840 (5)	0.58486 (5)	0.03957 (14)	
S21	0.60333 (4)	0.32092 (4)	0.36574 (4)	0.02772 (12)	
O1W	0.41649 (16)	0.13394 (15)	0.39419 (16)	0.0567 (5)	
H1WA	0.4000	0.1775	0.4391	0.085*	
H1WB	0.3648	0.0823	0.3752	0.085*	
C11	0.75308 (18)	−0.02181 (15)	0.17266 (16)	0.0271 (4)	
C12	0.97924 (18)	−0.02114 (17)	0.27670 (17)	0.0340 (5)	
H12	1.0649	−0.0297	0.3071	0.041*	
C13	0.91989 (16)	0.04749 (15)	0.31994 (16)	0.0260 (4)	
C14	0.97254 (16)	0.12436 (16)	0.41806 (16)	0.0258 (4)	
C15	1.09151 (19)	0.15449 (17)	0.47761 (19)	0.0357 (5)	
H15	1.1574	0.1284	0.4630	0.043*	
C16	0.94546 (18)	0.24140 (16)	0.54155 (16)	0.0297 (4)	
C21	0.72366 (16)	0.38186 (16)	0.22579 (16)	0.0283 (4)	
C22	0.68729 (19)	0.42159 (17)	0.32255 (18)	0.0351 (5)	
H22A	0.6365	0.4868	0.2955	0.042*	
H22B	0.7618	0.4418	0.3908	0.042*	
C23	0.45736 (17)	0.21590 (16)	0.14957 (16)	0.0281 (4)	
C24	0.46281 (18)	0.31021 (17)	0.23123 (18)	0.0331 (4)	
H24A	0.3931	0.3032	0.2522	0.040*	
H24B	0.4521	0.3782	0.1879	0.040*	

### Atomic displacement parameters (Å^2^)

**Table:**

	*U*^11^	*U*^22^	*U*^33^	*U*^12^	*U*^13^	*U*^23^
Ni	0.02234 (14)	0.02582 (15)	0.02243 (14)	0.00214 (9)	0.00973 (10)	−0.00135 (9)
O21	0.0427 (8)	0.0327 (8)	0.0364 (8)	0.0069 (6)	0.0245 (7)	0.0029 (6)
O22	0.0365 (7)	0.0379 (8)	0.0385 (8)	0.0002 (6)	0.0191 (6)	0.0072 (7)
O23	0.0279 (7)	0.0337 (8)	0.0318 (7)	0.0052 (6)	0.0074 (6)	−0.0068 (6)
O24	0.0369 (8)	0.0446 (9)	0.0313 (8)	0.0066 (7)	0.0005 (7)	−0.0055 (7)
O1	0.0366 (7)	0.0374 (8)	0.0335 (8)	−0.0030 (6)	0.0162 (6)	0.0014 (6)
N11	0.0262 (8)	0.0248 (8)	0.0228 (8)	0.0027 (6)	0.0109 (6)	0.0005 (6)
N12	0.0359 (9)	0.0368 (10)	0.0322 (9)	−0.0008 (8)	0.0100 (8)	−0.0114 (7)
N13	0.0250 (8)	0.0302 (9)	0.0223 (8)	−0.0005 (6)	0.0100 (7)	−0.0013 (6)
N14	0.0450 (10)	0.0488 (11)	0.0376 (10)	−0.0144 (9)	0.0229 (9)	−0.0197 (9)
S11	0.0450 (3)	0.0377 (3)	0.0358 (3)	0.0105 (2)	0.0213 (2)	−0.0049 (2)
S12	0.0302 (3)	0.0519 (3)	0.0318 (3)	−0.0123 (2)	0.0082 (2)	−0.0044 (2)
S21	0.0294 (3)	0.0303 (3)	0.0257 (2)	0.00101 (19)	0.0137 (2)	−0.00348 (19)
O1W	0.0560 (10)	0.0546 (10)	0.0718 (12)	−0.0176 (9)	0.0388 (10)	−0.0175 (9)
C11	0.0357 (10)	0.0229 (10)	0.0255 (9)	0.0026 (8)	0.0157 (8)	0.0020 (7)
C12	0.0307 (10)	0.0388 (11)	0.0338 (11)	0.0095 (9)	0.0147 (8)	0.0016 (9)
C13	0.0265 (9)	0.0284 (10)	0.0240 (9)	0.0044 (8)	0.0113 (8)	0.0057 (8)
C14	0.0237 (9)	0.0295 (10)	0.0252 (9)	0.0028 (7)	0.0113 (8)	0.0063 (8)
C15	0.0281 (10)	0.0443 (13)	0.0348 (11)	0.0006 (9)	0.0132 (9)	0.0017 (9)
C16	0.0326 (10)	0.0332 (11)	0.0232 (10)	−0.0061 (8)	0.0117 (8)	0.0004 (8)
C21	0.0195 (9)	0.0342 (11)	0.0289 (10)	−0.0007 (8)	0.0078 (8)	0.0019 (8)
C22	0.0404 (11)	0.0297 (11)	0.0383 (12)	−0.0026 (9)	0.0194 (9)	−0.0029 (9)
C23	0.0247 (9)	0.0308 (11)	0.0293 (10)	−0.0009 (8)	0.0118 (8)	−0.0012 (8)
C24	0.0280 (10)	0.0344 (11)	0.0334 (11)	0.0056 (8)	0.0091 (9)	−0.0039 (9)

### Geometric parameters (Å, °)

**Table:**

Ni—O1	2.0763 (14)	N14—H14B	0.8401
Ni—O21	2.0944 (14)	S11—C12	1.730 (2)
Ni—O23	2.0357 (14)	S11—C11	1.7434 (19)
Ni—N11	2.0634 (15)	S12—C15	1.721 (2)
Ni—N13	2.1094 (16)	S12—C16	1.734 (2)
Ni—S21	2.4461 (7)	S21—C22	1.800 (2)
O21—C21	1.253 (2)	S21—C24	1.813 (2)
O22—C21	1.249 (2)	O1W—H1WA	0.8530
O23—C23	1.262 (2)	O1W—H1WB	0.8413
O24—C23	1.244 (2)	C12—C13	1.344 (3)
O1—H1A	0.8465	C12—H12	0.9300
O1—H1B	0.7947	C13—C14	1.462 (3)
N11—C11	1.323 (2)	C14—C15	1.343 (3)
N11—C13	1.385 (2)	C15—H15	0.9300
N12—C11	1.323 (2)	C21—C22	1.523 (3)
N12—H12A	0.8078	C22—H22A	0.9700
N12—H12B	0.8657	C22—H22B	0.9700
N13—C16	1.313 (2)	C23—C24	1.522 (3)
N13—C14	1.391 (2)	C24—H24A	0.9700
N14—C16	1.353 (3)	C24—H24B	0.9700
N14—H14A	0.8259		
			
O23—Ni—N11	93.83 (6)	H1WA—O1W—H1WB	108.2
O23—Ni—O1	86.99 (6)	N12—C11—N11	124.86 (17)
N11—Ni—O1	97.92 (6)	N12—C11—S11	121.70 (15)
O23—Ni—O21	88.66 (6)	N11—C11—S11	113.42 (14)
N11—Ni—O21	89.21 (6)	C13—C12—S11	110.28 (15)
O1—Ni—O21	171.88 (5)	C13—C12—H12	124.9
O23—Ni—N13	173.36 (6)	S11—C12—H12	124.9
N11—Ni—N13	79.53 (6)	C12—C13—N11	115.81 (17)
O1—Ni—N13	94.23 (6)	C12—C13—C14	128.72 (17)
O21—Ni—N13	90.88 (6)	N11—C13—C14	115.46 (15)
O23—Ni—S21	84.09 (4)	C15—C14—N13	115.59 (18)
N11—Ni—S21	170.43 (4)	C15—C14—C13	128.58 (18)
O1—Ni—S21	91.31 (4)	N13—C14—C13	115.80 (15)
O21—Ni—S21	81.42 (4)	C14—C15—S12	110.29 (16)
N13—Ni—S21	102.40 (5)	C14—C15—H15	124.9
C21—O21—Ni	122.59 (12)	S12—C15—H15	124.9
C23—O23—Ni	120.86 (12)	N13—C16—N14	123.90 (18)
Ni—O1—H1A	108.8	N13—C16—S12	114.04 (14)
Ni—O1—H1B	116.4	N14—C16—S12	122.03 (15)
H1A—O1—H1B	106.3	O22—C21—O21	124.33 (18)
C11—N11—C13	111.02 (15)	O22—C21—C22	116.75 (18)
C11—N11—Ni	133.97 (12)	O21—C21—C22	118.89 (17)
C13—N11—Ni	114.65 (12)	C21—C22—S21	113.36 (14)
C11—N12—H12A	116.9	C21—C22—H22A	108.9
C11—N12—H12B	115.7	S21—C22—H22A	108.9
H12A—N12—H12B	127.3	C21—C22—H22B	108.9
C16—N13—C14	110.52 (16)	S21—C22—H22B	108.9
C16—N13—Ni	134.78 (13)	H22A—C22—H22B	107.7
C14—N13—Ni	111.95 (12)	O24—C23—O23	124.30 (18)
C16—N14—H14A	119.4	O24—C23—C24	115.78 (17)
C16—N14—H14B	115.9	O23—C23—C24	119.87 (16)
H14A—N14—H14B	114.5	C23—C24—S21	116.29 (13)
C12—S11—C11	89.46 (9)	C23—C24—H24A	108.2
C15—S12—C16	89.49 (10)	S21—C24—H24A	108.2
C22—S21—C24	100.37 (10)	C23—C24—H24B	108.2
C22—S21—Ni	94.66 (7)	S21—C24—H24B	108.2
C24—S21—Ni	94.68 (7)	H24A—C24—H24B	107.4

### Hydrogen-bond geometry (Å, °)

**Table:**

*D*—H···*A*	*D*—H	H···*A*	*D*···*A*	*D*—H···*A*
O1—H1A···O1W	0.85	1.91	2.7540	173
O1—H1B···O22^i^	0.79	1.97	2.7399	162
N12—H12A···O24^ii^	0.81	2.09	2.8785	166
N12—H12B···O23	0.87	2.09	2.8807	152
N14—H14A···O21^i^	0.83	2.20	2.9378	149
O1W—H1WA···O24^i^	0.85	2.01	2.8616	177
O1W—H1WB···O22^iii^	0.84	1.91	2.7493	173

Symmetry codes: (i) *x*, −*y*+1/2, *z*+1/2; (ii) −*x*+1, −*y*, −*z*; (iii) −*x*+1, *y*−1/2, −*z*+1/2.
